# Neutrophil-derived microparticles are released into the coronary circulation following percutaneous coronary intervention in acute coronary syndrome patients

**DOI:** 10.1042/BSR20160430

**Published:** 2017-01-17

**Authors:** Gonzalo J. Martínez, Jennifer Y. Barraclough, Shirley Nakhla, Vivian Kienzle, Stacy Robertson, Ziad Mallat, David S. Celermajer, Sanjay Patel

**Affiliations:** 1 Department of Cardiology, Royal Prince Alfred Hospital, Sydney, New South Wales, Australia; 2 Sydney Medical School, The University of Sydney, New South Wales, Australia; 3 División de Enfermedades Cardiovasculares, Pontificia Universidad Católica de Chile, Santiago, Chile; 4 Heart Research Institute, Sydney, New South Wales, Australia; 5 Institut National de la Santé et de la Recherche Médicale (INSERM), Paris Cardiovascular Research Center, Paris, France; 6 Division of Cardiovascular Medicine, University of Cambridge, Addenbrooke’s Hospital, Cambridge, United Kingdom

**Keywords:** Microparticles, Neutrophils, Acute Coronary Syndromes

## Abstract

To evaluate (i) local coronary and systemic levels of microparticles (MP) in acute coronary syndrome (ACS) and stable angina pectoris (SAP) patients and (ii) their release after plaque disruption with percutaneous coronary intervention (PCI). MP are small vesicles originating from plasma membranes of cells after activation or apoptosis and are implicated in the pathogenesis of atherosclerosis. Neutrophils play a role in plaque destabilization and shed neutrophil-derived MP that have the potential to drive significant proinflammatory and thrombotic downstream effects. Eight ACS and eight SAP patients were included. Coronary sinus (CS) samples pre-intervention (CS1), 45 s following balloon angioplasty (CS2) and at 45 s intervals following stent deployment (CS3, CS4 and CS5), together with peripheral vein samples, pre- and post-PCI were analysed for neutrophil-derived (CD66b+), endothelial-derived (CD144+), platelet-derived (CD41a+), monocyte-derived (CD14+) and apoptotic (Annexin V+) MP. ELISA for interleukin (IL)-6, myeloperoxidase (MPO) and P-selectin was also performed. CD66b+ MP levels were similar in both groups pre-intervention. Post-PCI, CS levels rose significantly in ACS but not SAP patients (ACS area under the curve (AUC): 549 ± 83, SAP AUC: 24 ± 29, *P*<0.01). CS CD41a+, CD144+, CD14+ and Annexin V+ MP levels did not differ between groups. Acute neutrophil-derived MP release post-PCI occurs in ACS compared with stable patients, likely to be reflective of plaque MP content in vulnerable lesions.

## Introduction

Microparticles (MP) are small vesicles (0.1–1 μm) originating from plasma membranes of cells. MP are most commonly derived from endothelial cells, platelets, leucocytes [1] and erythrocytes after activation or during early apoptosis [2,3]. They express surface antigens derived from their cell of origin, allowing for identification of subtype, and the majority (80%) of circulating MP are derived from platelets, with leucocyte-derived MP accounting for <10% [1,3]. MP play an active role in homoeostasis, inflammation, thrombosis, endothelial function, cell apoptosis, vascular remodelling and signal amplification, depending on their cell of origin [4].

MP are implicated in the pathogenesis of atherosclerosis; they are markedly increased in those with cardiovascular risk factors and in patients with subclinical atherosclerosis [1,5]. Moreover, *ex vivo* studies in human carotid plaque correlated the presence of leucocyte MP with plaque instability [6], whereas peripheral levels of endothelial-derived MP have been shown to be elevated in acute coronary syndrome (ACS) compared in patients with stable angina pectoris (SAP) [7]. However, characterization at local coronary level of leucocyte and particularly neutrophil-derived MP in patients with stable and unstable coronary plaque has not yet been described. Therefore, we aimed to evaluate (i) local coronary and systemic levels of MP in ACS and SAP patients and (ii) their release after percutaneous coronary intervention (PCI).

## Materials and methods

### Patient population

Sixteen consenting patients (eight ACS and eight SAP, as per ACC/AHA 2007 guidelines) [8] were recruited from the Department of Cardiology, Royal Prince Alfred Hospital (RPAH), Sydney, NSW, Australia. In all patients, the culprit lesion deemed appropriate for PCI was >70% stenosis.

### Blood sampling

**Figure 1:**
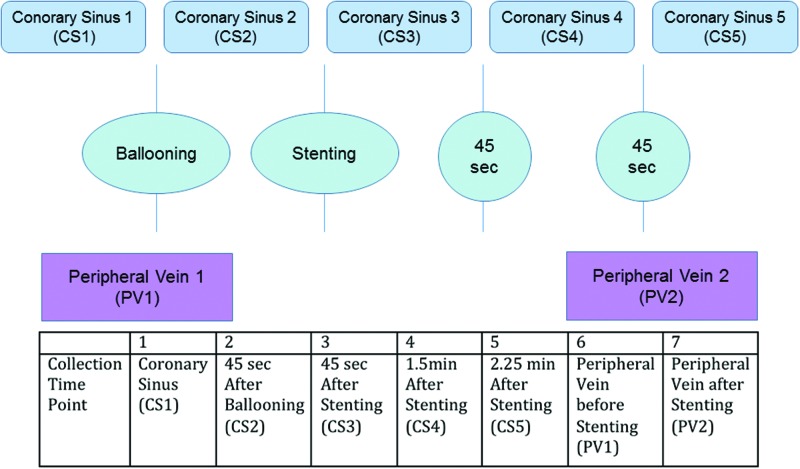
MP sampling protocol Simultaneous blood sampling from CS and PV pre-intervention (CS1 and PV1) followed by CS sampling 45 s after balloon angioplasty (CS2) was performed. After coronary artery stenting, CS sampling at 45 s intervals was performed (CS3, CS4 and CS5). A final venous sample (PV2) was drawn simultaneously with CS5.

During PCI, coronary sinus (CS) blood samples were collected at five intraprocedural time points (CS1–5). Peripheral venous (PV) blood was collected, pre-procedure (PV1) and post-procedure (PV2) from the common femoral vein (Figure 1). Our technique to safely cannulate the CS ostium has recently been described [9]. All blood samples were stored at ambient temperature and immediately processed within an hour of collection. Inclusion criteria included all patients >21 years of age who had a clinical indication for a coronary angiogram and PCI at RPAH. Exclusion criteria included patients with >50% stenosis in the left main coronary artery, cardiogenic shock or haemodynamic instability, age <21 years, pregnant or lactating women, moderate renal or hepatic dysfunction, thrombocytopenia or leucopenia. Patients with evidence of active infection or inflammatory conditions that might be associated with markedly elevated C-reactive protein (CRP) levels in the blood and those taking other anti-inflammatory therapies (e.g. corticosteroids) were also excluded. All patients received unfractionated heparin at a dose to achieve an activated clotting time of >250 s.

### Blood processing

Plasma was separated from whole blood by centrifugation at 20°C at 1500×***g*** for 10 min. A 300 μl volume of plasma was retained for additional differential centrifugation. Remaining plasma was stored at −80°C. Cell-derived MP were isolated from fresh plasma by differential centrifugation at 20°C at 12000×***g*** for 12 min. A volume of platelet-poor plasma (PPP) was retained for immediate antibody staining and flow cytometry (FCM) analysis.

### MP FCM

**Figure 2:**
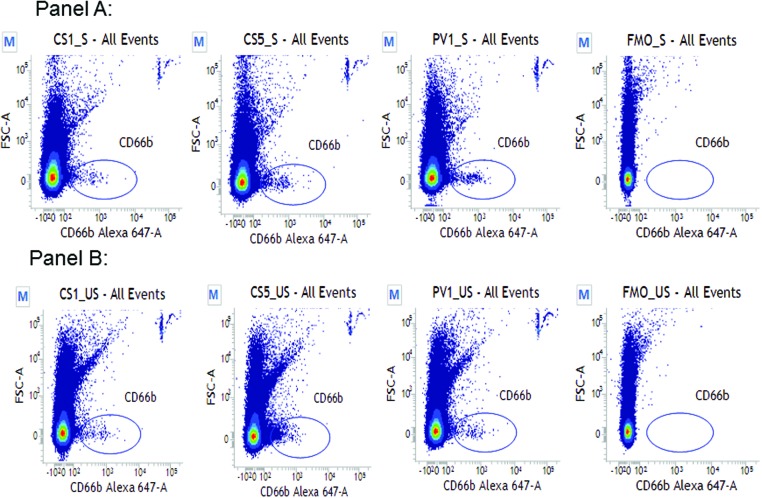
CD66b+ MP isolation by FCM Representative readouts of CD66b+ MP assessed by forward and side scatter after fluorochrome staining; exponential scale used, gated to size range 0.3–1 μm. Panel A: MP events seen within the gate in a representative SAP patient at CS1, CS5, PV1 and FMO (a negative control analysis). Panel B: MP events seen within the gate in a representative ACS patient at CS1, CS5, PV1 and FMO.

Blood sample collected was quantified with FCM (FACSVerse, BD Biosciences) and analysed using FACSuite software (v1.0.5.3841) (Figure 2). Based on the use of fluorescent-calibrated submicrometer beads (Megamix-Plus FSC (Nr.01077) and Megamix-Plus SSC (Nr.01078); BioCytex) size-related issues, resolution, PMT voltages and thresholds were optimized to cover a size-range from 0.3 to 1.0 μm MP equivalents. Spectral overlap among seven fluorochromes (FITC, BV421, BV510, PE-Cy-7, PE, AF647 and PerCP-5.5) was avoided by compensation using single-stained compensation beads (BD552843) and fluorescence minus one (FMO) controls. Mouse anti-human monoclonal antibodies (BD Biosciences) were used to quantify MP subtypes. Titration curves for all antibodies were used to establish appropriate antibody concentrations. Standardized FCM protocols were adapted and applied to all patient samples. MP in 20 μl of PPP were labelled with 100 μl antibody cocktail containing PBS, Annexin V–FITC (BD556419), CD14–BV421 (BD563743), CD31–BV510 (BD563454), CD33–PE-Cy7 (BD333946), CD41a–PE (BD555467), CD66b–AF647 (BD561645) and CD144–PerCP-5.5 (BD561566) at an ambient temperature for 10 min. After antibody labelling, MP were immediately analysed by FCM. MP subsets assessed included neutrophil-derived (CD66b+), endothelial-derived (CD144+), platelet-derived (CD41a+), monocyte-derived (CD14+) and apoptotic (Annexin V+). A low flow rate was used to acquire a total of 500000 events per sample. Trucount tubes were used to evaluate absolute MP counts. Results were expressed at events per microlitre [10,11].

### Plasma ELISA

Frozen plasma samples were assayed using ELISA to measure concentrations of interleukin (IL)-6 (R&D Systems Il-6 Quantikine ELISA kit), myeloperoxidase (MPO) (Hycult Biotech, Human MPO ELISA) and P-selectin (R&D Systems P-selectin/CD62P Quantikine) as per manufacturers’ instructions.

### Angiographic analysis

Lesion percentage stenosis and characteristics [12], stent length, size, number and type were recorded. Thrombosis in myocardial infarction (TIMI) flow post-intervention was documented [13]. Gensini score, a marker of atherosclerotic burden, determined angiographically incorporating the severity and location of all stenoses in the coronary bed, was also assessed pre-intervention [14].

### Statistical analysis

All continuous variables were reported as mean ± S.D. and categorical variables as number (percentage). Release of MP and cytokines over all sampling time points after PCI was quantified by area under the curve (AUC) ± S.E.M. Total AUC for each group or levels at specific time points between and within groups were compared using Student’s *t *test continuous variable associations, including Gensini score and MP levels were assessed with linear regression. All tests were two-tailed with acceptable type 1 error set at *P*<0.05. Statistical analyses were performed in Graph Pad Prism Version 6.0 (Graph Pad Software, LA Jolla, CA) and SPSS for Macintosh version 23 (IBM, Armonk, NY).

## Results

### Patient characteristics

Baseline characteristics were similar between groups (Table 1). Within the ACS group, four patients presented with an NSTEMI, two with STEMI with delayed revascularization and two with unstable angina pectoris (UAP). All patients received dual antiplatelet therapy pre-intervention and 11/16 received statin therapy at the time of intervention. Two patients in the ACS group received GpIIb/IIIa inhibitor during PCI and all patients received heparin pre-intervention, aiming for an activated clotting time of 250–300 s. CRP and troponin levels pre- and post-intervention were higher in the ACS group; however, absolute neutrophil count was similar between groups.

**Table 1:** Baseline characteristics of SAP and ACS patients

Baseline characteristics	Stable (*n*=8)	ACS (*n*=8)	*P* value
Age	68 (9)	65 (14)	0.56
Sex (Male)	7 (87.5%)	8 (100%)	1
**Cardiovascular risk factors**			
Hypertension	5 (62.5%)	7 (87.5%)	0.57
Hypercholesterolaemia	5 (62.5%)	6 (75%)	0.32
Diabetes	4 (50%)	2 (25%)	0.61
Smoker: past or present	5 (62.5%)	6 (75%)	1
Family history CAD	1 (12.5%)	3 (37.5%)	0.57
Renal impairment (GFR<60)	2 (25%)	1 (12.5%)	1
**Previous CAD**			
Previous revascularization	1 (12.5%)	4 (50%)	0.28
Previous AMI	0	3 (37.5%)	0.2
**Presentation**			
SAP	8 (100%)	0	
UAP	0	2 (25%)	
AMI	0	6 (75%)	
**Medications pre-procedure**			
Aspirin	8 (100%)	8 (100%)	1
Thienopyridines	8 (100%)	8 (100%)	0.47
B-blocker	3 (37.5%)	7 (87.5%)	0.12
Statin	5 (62.5%)	6 (75%)	1
ACEI/ARB	4 (50%)	7 (87.5%)	0.28
**Blood pre-PCI**			
CRP	2.77 (2.76)	24.35 (44.90)	0.35
hsTroponin	13.40 (10.31)	553.14 (653.30)	0.09
CK	136.60 (118.27)	297.17 (213.65)	0.17
WCC	7.85 (5.68)	8.95 (2.34)	0.62
Neutrophils	3.81 (1.87)	5.61 (1.85)	0.07
**Blood post-PCI**			
CRP	3	89 (105.64)	0.62
hsTroponin	215.00 (375.35)	440.57 (742.61)	0.46
CK	105.71 (49.50)	182.17 (133.38)	0.19

Values as mean ± S.D. or number (percentage). *P* value as Fisher’s exact test for categorical and Student’s *t* test for continuous variables. Abbreviations: ACEI/ARB, ace inhibitor/angiotensin receptor blocker; AMI, acute myocardial infarction; CABG, coronary artery bypass graft; CAD, coronary artery disease; CK, creatinine kinase; HDL, high density lipoprotein; hsTroponin, high sensitivity troponin; LDL, low density lipoprotein; WCC, white cell count.

### Angiographic characteristics

The mean stent length and diameter was 25.00 ± 15.05 mm and 3.18 ± 0.53 mm respectively in the SAP group and 20.25 ± 11.95 mm and 3.00 ± 0.53 respectively in the ACS group. All ACS patients and six out of eight SAP patients received drug-eluting stents (DESs), one SAP patient received a bioabsorbable scaffold and two received bare-metal stents (BMSs). TIMI frame count (TFC) was 16.4 ± 7.47 in SAP and 17.1 ± 8.26 in ACS, *P*=0.88. The Gensini score, a measure of extent of coronary atherosclerosis on angiography was similar in SAP and ACS patients (23.13 ± 19.74 compared with 41.38 ± 41.61, *P*=0.28) (Table 2).

**Table 2:** Baseline angiographic characteristics of SAP and ACS patients

Angioplasty characteristics	Stable **(***n*=8)	ACS (*n*=8)	*P* value
**Coronary artery for PCI**			0.61
LAD	4 (50%)	2 (25%)	
Lcx	0	1 (12.5%)	
RCA	4 (50%)	5 (62.5%)	
**Number of vessel disease(s)**			1
3	1 (12.5%)	2 (25%)	
2	3 (37.5%)	3 (37.5%)	
1	4 (50%)	3 (37.5%)	
ACC/AHA lesion type*			1
A	1 (12.5%)	0	
B	5 (62.5%)	5 (62.5%)	
C	2 (25%)	3 (37.5%)	
**Lesion characteristics**			
CTO	2 (25%)	0	
Length (mm)	12.8 (5.9)	16.6 (9.2)	0.39
100% stenosis	2 (25%)	0	
**TIMI flow grade**			
TIMI 3	7 (87.5%)	8 (100%)	
TIMI 2	1 (12.5%)	0	
TFC	16.47 (7.47)	17.14 (8.26)	0.88
Gensini score	23.13 (19.74)	41.38 (41.61)	0.28
**Stent characteristics**			
Length (mm)	25.00 (15.05)	20.25 (11.95)	0.59
Size	3.18 (0.53)	3.00 (0.53)	0.23
**Stent type**			0.47
DES	6 (75%)	8 (100%)	
BMS	1 (12.5%)	0	
Bioabsorbable scaffold	1 (12.5%)	0	
**Time to PCI from presentation in ACS**			
<24 h	-	1 (12.5%)	
<48 h	-	1 (12.5%)	
>48 h	-	4 (50%)	
N/A (UAP)	-	2 (25%)	

Values as mean ± S.D. or number (percentage). *P* value as Fisher’s exact test for categorical and Student’s *t* test for continuous variables. Abbreviations: CTO, chronic total occlusion; LAD, left anterior descending; Lcx, left circumflex; RCA, right coronary artery; TIMI flow grade and frame count; *–Refers to treated lesion.

### Neutrophil-derived MP

**Figure 3:**
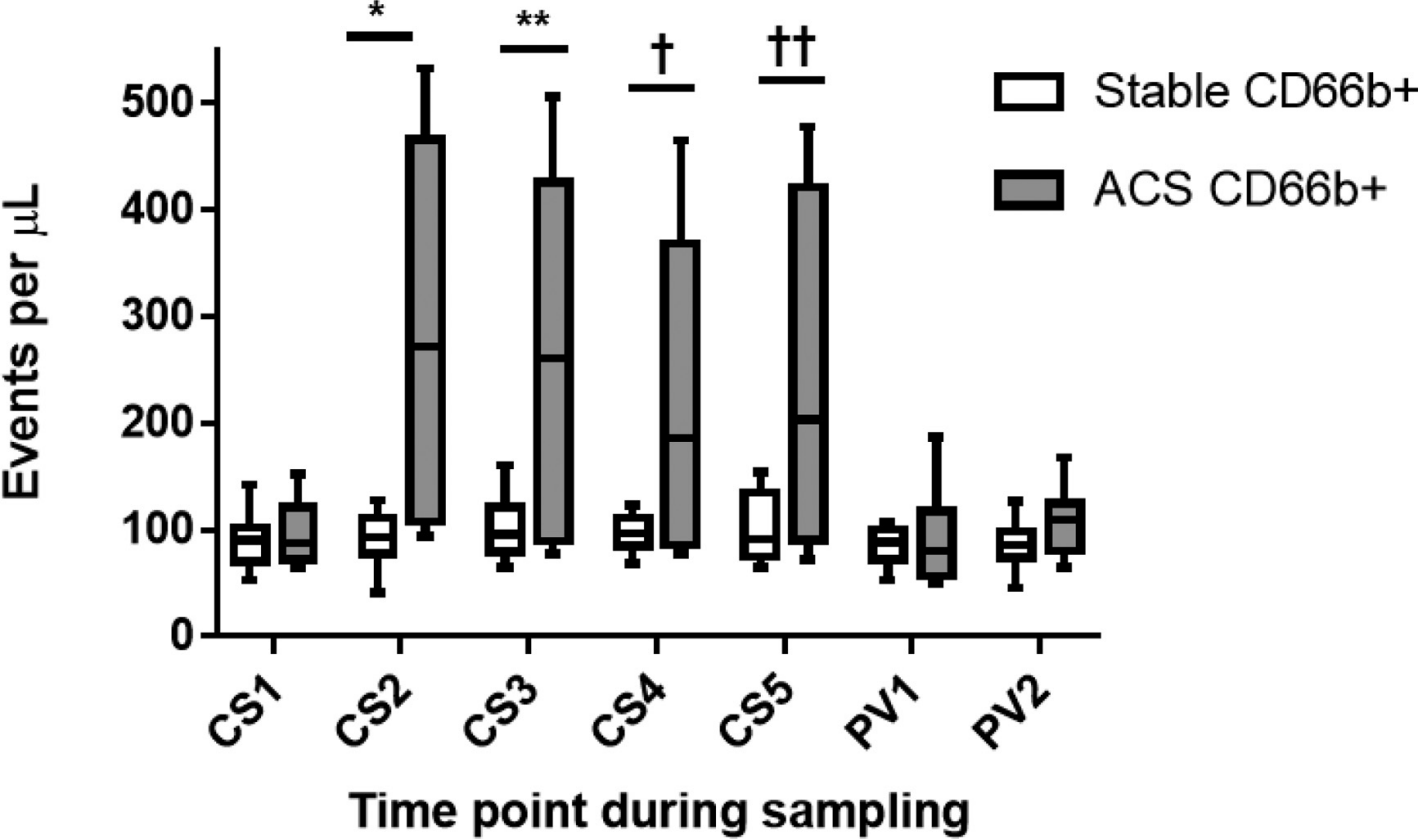
CD66b+ MP release after PCI in SAP and ACS patients CS and peripheral vein (PV) samples were drawn from SAP (*n*=8) and ACS patients (*n*=8) pre- and post-PCI. Results as median, 25th and 75th percentiles and minimum and maximum error bars. CS1 and PV1–pre-intervention, CS2-5 and PV2 post-intervention as described in Figure 1. *CS2 SAP compared with CS2 ACS; *P*=0.009, **CS3 SAP compared with CS3 ACS; *P*=0.02, ^†^CS4 SAP compared with CS4 ACS; *P*=0.03, ^††^CS5 SAP compared with CS5 ACS; *P*=0.03.

Pre-intervention CD66b+ MP CS1 and PV1 levels were comparable between groups and within each group there was no difference in CS and PV levels. Post-intervention, CS levels increased significantly in ACS compared with SAP patients (ACS AUC: 549 ± 83, SAP AUC: 24 ± 29, *P*=0.01). Levels of this MP were also significantly higher in ACS compared with SAP patients at each time point post-intervention (CS2: ACS 278.80 ± 61.71 events/μl compared with SAP 90.98 ± 9.27 events/μl; *P*=0.009; CS3: ACS 264.10 ± 62.90 events/μl compared with SAP 101.30 ± 10.83 events/μl; *P*=0.02; CS4: ACS 224.50 ± 53.09 events/μl compared with SAP 97.67 ± 6.26 events/μl; *P*=0.03; CS5: ACS 246.30 ± 58.33 events/μl compared with SAP 102.30 ± 11.56 events/μl; *P*=0.03) (Figure 3). Post-intervention, PV were comparable to pre-PCI levels in both groups.

### Platelet-derived MP

**Figure 4:**
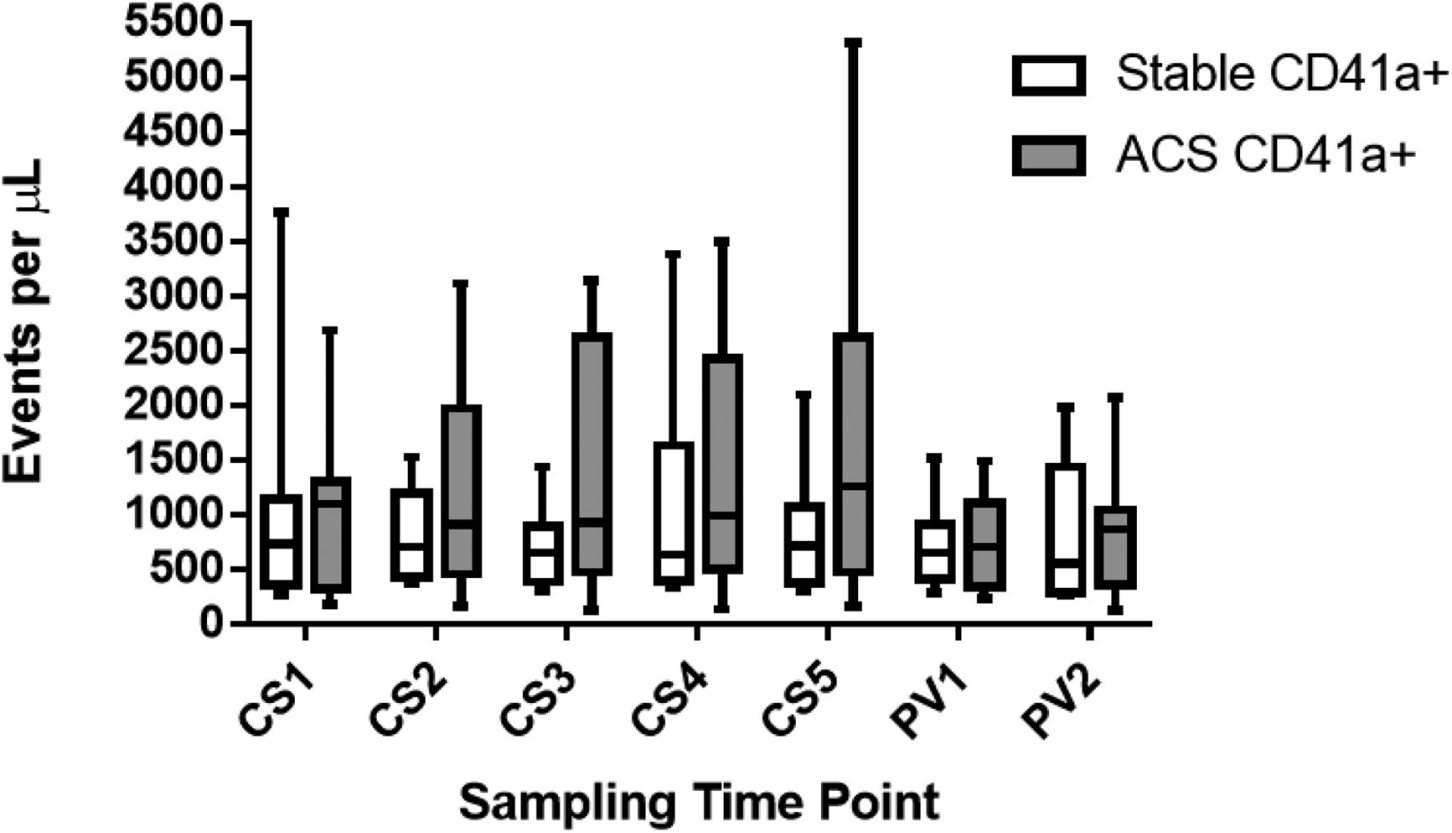
CD41a+ MP release after PCI from SAP and ACS patients CD41a+ MP from stable CAD (*n*=8) and ACS patients (*n*=8) pre- and post-intervention in the CS and PV circulation. Results as median, 25th and 75th percentiles and minimum and maximum error bars as events per microlitre (μl). Time points CS1 and PV1–pre-intervention, CS2-5 and PV2 post-intervention as described in Figure 1. CS1 ACS v CS5 ACS; *P*=0.08.

Pre-intervention, platelet-derived MP levels (CD41a+) in PV and CS blood were comparable between groups. Post-intervention, CS levels did not increase in ACS compared with SAP patients (SAP AUC: 646 ± 949, ACS AUC: 1115 ± 526, *P*=0.12). There was a non-significant trend to increased levels in ACS patients at CS5 time point (1757 ± 1672 events/μl) compared with CS1 (1053 ± 791.8 events/μl) in the ACS group (*P*=0.08). Post-intervention, PV levels were comparable to pre-PCI levels in both groups (Figure 4).

### Endothelial, monocyte and apoptotic MP

Endothelial-derived MP (CD144+ MP), monocyte-derived MP (CD14+ MP) and apoptotic (Annexin V+) MP levels were not significantly different than pre-intervention in either group and were unaffected by PCI.

### Proportions of MP pre- and post-intervention

**Figure 5:**
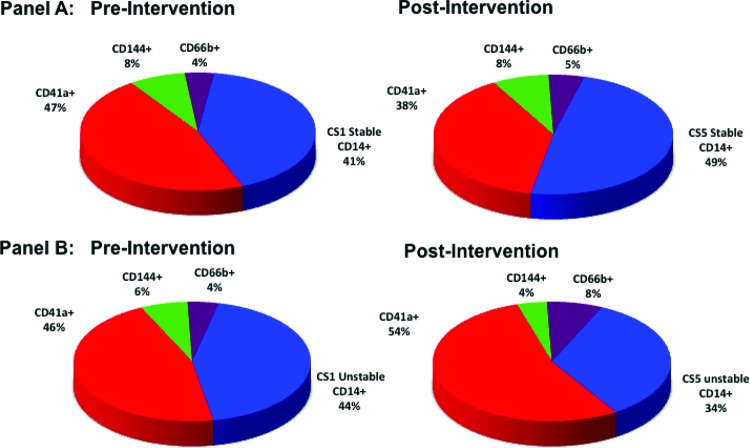
Distribution of MP by subtype in the CS before and after PCI in patients with SAP and ACS Panel A: Pie-charts showing distribution in CS blood of MP populations from SAP patients (*n*=8) pre-PCI (left) and post-PCI (right) represented as percent of total MP. Panel B: Pie-charts showing distribution of MP in CS blood from ACS patients (*n*=8) pre-intervention (left) and post-intervention (right) represented as percent of total MP

Pre-intervention platelet-derived MP accounted for the majority of MP levels detected followed by monocytes/macrophages and to a significantly smaller degree neutrophil and endothelial-derived MP. Post-PCI levels of neutrophil-derived and platelet-derived MP increased in ACS but not in SAP patients (Figure 5).

### Cytokine levels

#### IL-6

Pre-intervention, CS IL-6 levels appeared numerically but not statistically and significantly higher in ACS compared with SAP patients (83.8 ± 40.6 pg/ml compared with 5.4 ± 1.8 pg/ml, *P*=0.07). Post-intervention, CS analysis again demonstrated no significant difference despite numerically greater AUC in ACS compared with SAP patients (23.3 ± 11.6 compared with 1.4 ± 1.4, *P*=0.08). In both groups, there were no significant differences in venous levels pre- and post-intervention (SAP pre: 7.2 ± 2.1 pg/ml, SAP post: 7.6 ± 2.3 pg/ml; ACS pre: 49.3 ± 22.5 pg/ml, ACS post: 52.2 ± 25 pg/ml).

#### MPO

Pre-intervention, CS levels were comparable in both patient groups (SAP: 135.9 ± 8.1 ng/ml, ACS: 128.5 ± 21.2 ng/ml). Post-intervention, there was a significantly greater AUC in ACS compared with SAP patients (71.2 ± 25.9 compared with 2.3 ± 9.8, *P*=0.03). In either patient group, venous levels were similar pre- and post-intervention (SAP pre: 140.0 ± 5.5 ng/ml, SAP post: 145.0 ± 6.7 ng/ml; ACS pre: 157.2 ± 7.9 ng/ml, ACS post: 169.2 ± 11.8 ng/ml).

#### P-selectin

Pre-intervention, CS levels were comparable in both patient groups (SAP: 115.5 ± 19.9 ng/ml, ACS: 128.3 ± 14.7 ng/ml), with a non-significant change in AUC post-intervention in ACS patients compared with SAP patients (unstable: 23.4 ± 21.5, stable: 0.3 ± 6.3, *P*=0.3). In either group, pre- and post-intervention venous levels were comparable (SAP pre: 123.2 ± 18 ng/ml, SAP post: 119.5 ± 18.3 ng/ml; ACS pre: 116.1 ± 16.4 ng/ml, ACS post: 116.5 ± 13.4 ng/ml).

Pre-intervention CD66b+ MP levels were significantly higher in 6/16 diabetics (*P*=0.037), however remained similar when analysed with respect to other cardiovascular risk factors, lesion length, complexity, overall coronary burden, stent length or pre-PCI troponin level. Post-intervention MP levels were not different when patients were analysed with respect to any of these factors.

## Discussion

Here, we demonstrate for the first time, striking differences in MP release following plaque disruption by PCI in ACS compared with SAP patients, with an early and highly significant increase in CD66b+ MP and a late increase in CD41a+ MP. Notably, these changes were only seen in the CS, underpinning the importance of intracardiac sampling as a more specific measure of the local coronary microenvironment compared with peripheral sampling alone. In parallel with CD66b+ release, we also demonstrate a significant release of MPO into the coronary circulation in ACS patients.

MP have previously been identified in human atherosclerotic plaque [15] and have been implicated in promoting plaque instability [6]. Moreover, MP analysis in STEMI patients revealed decreased concentrations of all MP, except for leucocyte-derived MP, which did not change [16]. Notably, in the present study, coronary artery blood distal to the stenosis was assayed, and it is therefore plausible that MP were rapidly washed-out into the distal circulation after coronary stenting and therefore could not be accurately measured. In contrast, our study employed CS sampling, which allowed us to measure local coronary changes more accurately and immediately after PCI as compared with PV or distal coronary artery sampling. Our study demonstrated a high concentration of CD66b+ MP released from unstable coronary plaque. Importantly, pre-PCI levels of these mediators were comparable between groups. There are three possible explanations for our findings: (i) CD66b+ MP were already shed by plaque-associated activated neutrophils before PCI, but trapped within plaque, possibly within thrombus, with PCI resulting in disruption of the plaque–thrombus interface and MP release into the coronary circulation, (ii) PCI induces *de novo* neutrophil activation – as plaque-trapped neutrophils are more abundant in vulnerable compared with stable plaques, PCI will generate greater release of CD66b+ MP from vulnerable plaque and (iii) PCI brings cholesterol crystals (which are more abundant in vulnerable compared with stable plaque) in close contact with blood neutrophils, which may lead to their activation [17].

Neutrophils are thought to play a key role in plaque destabilization [18]. Activated neutrophils shed CD66b+ MP, which might drive a number of acute inflammatory and thrombotic effects including direct activation of the complement cascade [19], activation of endothelial cells with inflammatory cytokine release and promotion of local tissue destruction via expression of MPO, elastase, MMP-9 or proteinase-3 [20]. Consistent with these findings, we also observed a parallel rise in MPO and IL-6 release after plaque disruption in ACS patients. MPO is a pro-atherogenic enzyme released from activated neutrophils [21], which promotes dissolution of the fibrous cap of plaque, leading to plaque rupture [22]. IL-6 is key downstream inflammatory cytokine, strongly linked with disease activity [23] as well as future coronary events [24,25]. Taken together, these effects underpin a potential pathogenic role for these MP in patients with plaque destabilization.

The consequences of CD66b+ MP release in the setting of PCI may also explain some of the complications seen with PCI in ACS but only rarely with SAP patients, such as no-reflow, thrombosis and microvascular dysfunction. Leucocyte-derived MP in atherosclerotic plaque promote tissue factor activation [4,26,27], which is implicated in coronary no-reflow [28]. Neutrophil activation and thrombosis may promote microvessel obstruction and increased infarct size via neutrophil plugging and microthrombi [29]. Indeed, a recent study that analysed coronary thrombus in the setting of ACS, demonstrated the presence of activated neutrophils, the levels of which correlated with infarct size [30]. In our study TFC, as assessed by standard methodology [13], was similar between groups and inconsistent with previous studies demonstrating platelet and endothelial-derived MP to be associated with microvascular obstruction as assessed by TFC [31]. However an alternate invasive assessment of microvascular function post-PCI (e.g. recording the index of microcirculatory resistance) [32] might demonstrate an association between release of CD66+ MP and microvascular dysfunction; this requires further study.

We also demonstrated a late non-significant rise in platelet-derived CD41a+ MP in ACS patients only, and it is conceivable that even later sampling may have yielded significant differences. In particular, Inoue et al. [33] demonstrated in stable patients undergoing LAD PCI, significant elevations in these MP at 24 and 48 h in CS and PV post-PCI. It is therefore plausible that this late rise in CD41a+ MP may represent activation of platelets by α_M_β_2_ expression [34] on CD66b+ MP, rather than release of intraplaque CD41a+ MP.

Limitations of our study include the small sample size and thus conclusions drawn on eight patients per group should be interpreted with care. This precluded multivariate analysis for confounding variables, due to the challenging nature of collecting multiple intracardiac samples, which were then immediately analysed. Nonetheless, we believe our FCM protocol offers several advantages over recently published MP studies, in that we used fresh not frozen blood, as the freeze–thaw process has been shown to deleteriously affect accurate identification of MP populations, as well as employing single markers to accurately detect a range of MP subpopulations [35]. Moreover, by taking multiple samples during PCI, we were able to acutely study the release kinetics of these MP. We did not assess release of the very small MP population (100–300 nm). FCM cannot reliably detect these particles and other techniques cannot reliably characterize them to a cell of origin, which was the primary aim of the present study. While we have documented the release of neutrophil-derived MP into the coronary circulation after plaque disruption, we cannot conclude that these MP directly contribute to intracardiac inflammation and post-PCI complications, which are both driven by multiple pathways [36]. Lastly, we did not observe an association between CD66b+ MP release and adverse outcomes, e.g. infarct size.

## Conclusion

In the present study we demonstrated, for the first time, in humans, *in vivo* evidence of neutrophil activation in vulnerable coronary plaque, as demonstrated by an acute release of neutrophil-derived CD66b+ MP and MPO after PCI, providing a unique insight into unstable plaque biology and inflammatory activation occurring in the coronary circulation after PCI of vulnerable plaque. Further studies are currently being conducted to address the possible physiologic consequences of this release, correlate plaque composition as determined by intravascular imaging with MP release and to assess whether MP might be a potential therapeutic target to reduce PCI complications in ACS patients.

## Abbreviations

ACS: acute coronary syndrome
AUC: area under the curve
BMS: bare-metal stent
CAD: coronary artery disease
CRP: C-reactive protein
CS: coronary sinus
DES: drug-eluting stent
FCM: flow cytometry
FMO: fluorescence minus one
GpIIb/IIIa: glycoprotein IIb/IIIa
IL: interleukin
LAD: left anterior descending
Lcx: left circumflex
MP: microparticles
MMP-9: matrix metalloproteinase 9
MPO: myeloperoxidase
NSTEMI: non-ST elevation myocardial infarction
PCI: percutaneous coronary intervention
PPP: platelet-poor plasma
PV: peripheral venous
RCA: right coronary artery
SAP: stable angina pectoris
STEMI: ST elevation myocardial infarction
TFC: TIMI frame count
TIMI: thrombosis in myocardial infarction
UAP: unstable angina pectoris

## References

[1] Angelillo-Scherrer A. (2012) Leukocyte-derived microparticles in vascular homeostasis. Circ. Res. 110, 356–369 22267840 10.1161/CIRCRESAHA.110.233403

[2] Chironi G., Simon A., Hugel B., Del Pino M., Gariepy J., Freyssinet J.M. (2006) Circulating leukocyte-derived microparticles predict subclinical atherosclerosis burden in asymptomatic subjects. Arterioscler. Thromb. Vasc. Biol. 26, 2775–2780 17038634 10.1161/01.ATV.0000249639.36915.04

[3] Suades R., Padro T., Alonso R., Lopez-Miranda J., Mata P. and Badimon L. (2014) Circulating CD45+/CD3+ lymphocyte-derived microparticles map lipid-rich atherosclerotic plaques in familial hypercholesterolaemia patients. Thromb. Haemost. 111, 111–121 24085382 10.1160/TH13-07-0612

[4] Owens, III A.P. and Mackman N. (2011) Microparticles in hemostasis and thrombosis. Circ. Res. 108, 1284–1297 21566224 10.1161/CIRCRESAHA.110.233056 PMC3144708

[5] Wang J.G., Aikawa E. and Aikawa M. (2013) Leukocyte-derived microparticles as proinflammatory mediators in atherosclerosis. J. Am. Coll. Cardiol. 62, 1442–1445 23707315 10.1016/j.jacc.2013.04.054

[6] Sarlon-Bartoli G., Bennis Y., Lacroix R., Piercecchi-Marti M.D., Bartoli M.A., Arnaud L. (2013) Plasmatic level of leukocyte-derived microparticles is associated with unstable plaque in asymptomatic patients with high-grade carotid stenosis. J. Am. Coll. Cardiol. 62, 1436–1441 23707318 10.1016/j.jacc.2013.03.078

[7] Mallat Z., Benamer H., Hugel B., Benesssiano J., Steg G., Freyssinet J.M. (2000) Elevated levels of shed membrane microparticles with procoagulant potential in the peripheral circulating blood of patients with acute coronary syndromes. Circulation 101, 841–843 10694520 10.1161/01.cir.101.8.841

[8] King, III S.B., Smith, Jr. S.C., Hirshfeld, Jr. J.W., Jacobs A.K., Morrison D.A., Wiliams D.O. (2008) 2007 focused update of the ACC/AHA/SCAI 2005 guideline update for percutaneous coronary intervention: a report of the American College of Cardiology/American Heart Association Task Force on practice guidelines. J. Am. Coll. Cardiol. 51, 172–209 18191745 10.1016/j.jacc.2007.10.002

[9] Martinez G.J., Bailey B.P., Celermajer D.S. and Patel S. (2014) A safe and easy technique to sample the coronary sinus–facilitating a closer look at cardiac disease. Int. J. Cardiol. 176, 1321–1322 25139322 10.1016/j.ijcard.2014.07.151

[10] Orozco A.F. and Lewis D.E. (2010) Flow cytometric analysis of circulating microparticles in plasma. Cytometry A. 77, 502–514 20235276 10.1002/cyto.a.20886 PMC2919894

[11] Gelderman M. and Simak J. (2008) Flow cytometric analysis of cell membrane microparticles. Methods Mol. Biol. 79–93 10.1007/978-1-59745-398-1_6 18592174

[12] Ryan T.J., Faxon D.P., Gunnar R.M., Kennedy J.W., King, III S.B., Loop F.D. (1988) Guidelines for percutaneous transluminal coronary angioplasty. A report of the American College of Cardiology/American Heart Association Task Force on assessment of diagnostic and therapeutic cardiovascular procedures (Subcommittee on percutaneous transluminal coronary angioplasty). Circulation 78, 486–502 2969312 10.1161/01.cir.78.2.486

[13] Gibson C.M., Cannon C.P., Daley W.L., Dodge, Jr. J.T., Alexander, Jr. B., Marble S.J. (1996) TIMI frame count: a quantitative method of assessing coronary artery flow. Circulation 93, 879–888 8598078 10.1161/01.cir.93.5.879

[14] Gensini G.G. (1983) A more meaningful scoring system for determining the severity of coronaory heart disease. Am. J. Cardiol. 51, 606 6823874 10.1016/s0002-9149(83)80105-2

[15] Leroyer A.S., Isobe H., Leseche G., Castier Y., Wassef M., Mallat Z. (2007) Cellular origins and thrombogenic activity of microparticles isolated from human atherosclerotic plaques. J. Am. Coll. Cardiol. 49, 772–777 17306706 10.1016/j.jacc.2006.10.053

[16] Min P.K., Kim J.Y., Chung K.H., Lee B.K., Cho M., Lee D.L. (2013) Local increase in microparticles from the aspirate of culprit coronary arteries in patients with ST-segment elevation myocardial infarction. Atherosclerosis 227, 323–328 23422831 10.1016/j.atherosclerosis.2013.01.032

[17] Warnatsch A., Ioannou M., Wang Q. and Papayannopoulos V. (2015) Inflammation. Neutrophil extracellular traps license macrophages for cytokine production in atherosclerosis. Science 349, 316–320 26185250 10.1126/science.aaa8064 PMC4854322

[18] Doring Y., Drechsler M., Soehnlein O. and Weber C. (2015) Neutrophils in atherosclerosis: from mice to man. Arterioscler. Thromb. Vasc. Biol. 35, 288–295 25147339 10.1161/ATVBAHA.114.303564

[19] Mesri M. and Altieri D.C. (1998) Endothelial cell activation by leukocyte microparticles. J. Immunol. 161, 4382–4387 9780216

[20] Gasser O., Hess C., Miot S., Deon C., Sanchez J.-C. and Schifferli J.A. (2003) Characterisation and properties of ectosomes released by human polymorphonuclear neutrophils. Exp. Cell Res. 285, 243–257 12706119 10.1016/s0014-4827(03)00055-7

[21] Baldus S., Heeschen C., Meinertz T., Zeiher A.M., Eiserich J.P., Munzel T. (2003) Myeloperoxidase serum levels predict risk in patients with acute coronary syndromes. Circulation 108, 1440–1445 12952835 10.1161/01.CIR.0000090690.67322.51

[22] Nicholls S.J., Hazen S.L. (2005) Myeloperoxidase and cardiovascular disease. Arterioscler. Thromb. Vasc. Biol. 25, 1102–1111 15790935 10.1161/01.ATV.0000163262.83456.6d

[23] Martinez G.J., Robertson S., Barraclough J., Xia Q., Mallat Z., Bursill C. (2015) Colchicine acutely suppresses local cardiac production of inflammatory cytokines in patients with an acute coronary syndrome. J. Am. Heart Assoc. 4, e002128 26304941 10.1161/JAHA.115.002128 PMC4599469

[24] Ikeda U., Ito T. and Shimada K. (2001) Interleukin-6 and acute coronary syndrome. Clin. Cardiol. 24, 701–704 11714126 10.1002/clc.4960241103 PMC6655151

[25] Kaptoge S., Seshasai S.R., Gao P., Freitag D.F., Butterworth A.S., Borglykke A. (2014) Inflammatory cytokines and risk of coronary heart disease: new prospective study and updated meta-analysis. Eur. Heart J. 35, 578–589 24026779 10.1093/eurheartj/eht367 PMC3938862

[26] Mallat Z., Hugel B., Ohan J., Leseche G., Freyssinet J.-M. and Tedgui A. (1999) Shed membrane microparticles with procoagulant potential in human atherosclerotic plaques: a role for apoptosis in plaque thrombogenicity. Circulation 99, 348–353 9918520 10.1161/01.cir.99.3.348

[27] Amabile N., Rautou P.E., Tedgui A. and Boulanger C.M. (2010) Microparticles: key protagonists in cardiovascular disorders. Semin.Thromb. Hemost. 36, 907–916 21069633 10.1055/s-0030-1267044

[28] Bonderman D., Teml A., Jakowitsch J., Adlbrecht C., Gyongyosi M., Sperker W. (2002) Coronary no-reflow is caused by shedding of active tissue factor from dissected atherosclerotic plaque. Blood 99, 2794–2800 11929768 10.1182/blood.v99.8.2794

[29] Ito H. (2006) No-reflow phenomenon and prognosis in patients with acute myocardial infarction. Nat. Clin. Pract. Cardiovasc. Med. 3, 499–506 16932767 10.1038/ncpcardio0632

[30] Mangold A., Alias S., Scherz T., Hofbauer T., Jakowitsch J., Panzenbock A. (2015) Coronary neutrophil extracellular trap burden and deoxyribonuclease activity in ST-elevation acute coronary syndrome are predictors of ST-segment resolution and infarct size. Circ. Res. 116, 1182–1192 25547404 10.1161/CIRCRESAHA.116.304944

[31] Porto I., Biasucci L.M., De Maria G.L., Leone A.M., Niccoli G., Burzotta F. (2012) Intracoronary microparticles and microvascular obstruction in patients with ST elevation myocardial infarction undergoing primary percutaneous intervention. Eur. Heart J. 33, 2928–2938 22453653 10.1093/eurheartj/ehs065

[32] Fearon W.F., Low A.F., Yong A.S., McGeoch R., Berry C., Shah M.G. (2013) Prognostic value of the index of microcirculatory resistance measured after primary percutaneous coronary intervention. Circulation 127, 2436–2441 23681066 10.1161/CIRCULATIONAHA.112.000298 PMC5429864

[33] Inoue T., Komoda H., Kotooka N., Morooka T., Fujimatsu D., Hikichi Y. (2008) Increased circulating platelet-derived microparticles are associated with stent-induced vascular inflammation. Atherosclerosis 196, 469–476 17234194 10.1016/j.atherosclerosis.2006.12.004

[34] Pluskota E., Woody N.M., Szpak D., Ballantyne C.M., Soloviev D.A., Simon D.I. (2008) Expression, activation, and function of integrin alphaMbeta2 (Mac-1) on neutrophil-derived microparticles. Blood 112, 2327–2335 18509085 10.1182/blood-2007-12-127183 PMC2532806

[35] Shantsila E., Montoro-Garcia S., Gallego P. and Lip G.Y. (2014) Circulating microparticles: challenges and perspectives of flow cytometric assessment. Thromb. Haemost. 111, 1009–1014 24553954 10.1160/TH13-11-0937

[36] Hansson G.K. and Hermannsson A. (2011) The immune system in atherosclerosis. Nat. Immunol. 12, 204–212 21321594 10.1038/ni.2001

